# The relationship between agility and lower extremity strength in female basketball players

**DOI:** 10.1186/s13102-025-01250-y

**Published:** 2025-07-12

**Authors:** Ali Kerim Yılmaz, Soner Akgün, Esra Korkmaz Salkılıç, Berna Anıl, Enes Akdemir, Burcu Aktaş, Emre Karaduman, Menderes Kabadayı

**Affiliations:** 1 Faculty of Yasar Doğu Sport Sciences, Ondokuz Mayıs University, Samsun, 55270 Turkey; 2 Faculty of Sports Sciences, Artvin Çoruh University, Artvin, 08100 Turkey; 3 Faculty of Sports Sciences, Manisa Celal Bayar University, Manisa, 45140 Turkey

**Keywords:** Agility, Hop tests, Isokinetic, 1 RM, Basketball

## Abstract

**Background:**

The aim of the study was to investigate the relationship between agility and lower extremity strength in female basketball players.

**Methods:**

Fifteen females aged 18–24 years (age 20.80 years, height 1.70 cm, weight 67.60 kg and body mass index (BMI) 23.20 kg/m²) participated in the study voluntarily. In the study, 5-0-5 Agility (505), Pro-Agility (PA), T Agility (T-test), T Drill Hop (T) tests were used to determine the agility skills of the participants. To determine the lower extremity strength performance of the participants, concentric/concentric (Con/Con) isokinetic knee extension (Ex) and flexion (Flx) tests at angular velocities of 60, 180, 240 and 300°/sec, 5 different single leg hop tests (SLHT) [single leg hop for distance (SL), triple leg hop for distance (THD), crossover hop for distance (CHD), medial side triple hop for distance (MSTH), 90° medial rotation hop for distance (MRH)] and 3 different one-repetition maximum (1 RM) [leg press (LP), leg extension (LEX) and leg curl (LC)] tests were performed.

**Results:**

The results of the study showed a moderate, high and excellent negative correlation between SLHTs and agility (*p* < 0.05). Comparisons of agility and strength parameters on the right (R_S_) and left (L_S_) sides showed no significant difference (*p* > 0.05). In addition, ipsilateral hamstring/quadriceps (H/Q) and bilateral (H/H-Q/Q) strength ratios and limb symmetry indexes (LSI) obtained from the strength results of the subjects were within the normal range.

**Conclusions:**

*As a result*,* we found a negative relationship between agility and lower extremity functional performance tests in female basketball players.* In addition, it can be said that participants not likely to be injured under normal conditions (non-contact) because of the asymmetric strength ratios are within the safe range.

## Introduction

Basketball, a globally popular and dynamic sport, is characterised by jumps, linear sprints, accelerations, decelerations and vertical or horizontal change of direction actions of various intensities and durations [1–3]. The organism needs optimisation of lower body physical components such as multi-directional running, strength, vertical jumping and agility to successfully perform these short-term and high-intensity actions [4, 5]. In a basketball competition, players tend to change their movement intensity and pattern every 2–3 s (997 ± 183 change of direction per game), requiring optimal effort with high levels of agility and strength to gain an advantage over their opponents [6, 7]. This reveals the importance of agility skills to adapt to the ever-changing dynamics of modern basketball [8].

Agility includes sudden changes of direction, acceleration and deceleration combined with mental acuity [9] and is a basic physiological requirement in basketball [10]. Successful execution of explosive movements requires not only agility but also optimal muscular strength and power [11]. Through the stretch-shortening cycle (SSC), the eccentric and concentric strengths of the quadriceps and hamstring muscles directly influence the change of direction performance [3, 12, 13]. Furthermore, it has been suggested that an increase in eccentric strength of the quadriceps can translate into effective braking ability and that neuromuscular adaptations - neural stimulation, rapid SSC, and muscle coordination - enhance change of direction performance [13, 14]. Similarly, studies have reported strong correlations between maximum power parameters and vertical jump (*r* = 0.64–0.74), horizontal jump (*r* = 0.67) and change of direction (*r* = 0.89) performances [15–17].

*Actions such as dunk*,* rebound and blocking*,* which are among the basic movements of the basketball game*,* require high jumping. In these movements*,* the lower extremity muscles (especially the quadriceps*,* hamstring*,* gluteal and gastrocnemius groups) should be strong and monitored regularly with highly reliable measurement tools* [4–11]. Currently, isokinetic dynamometers are the most valid and reliable method for measuring lower extremity strength and power potential [18]. This method not only determines the strength and power potential of athletes, but also detects bilateral strength asymmetries that lead to performance differences and injury risk [19–21]. However, the functionality of isokinetic measurements is limited since they are performed at a single joint and constant velocity, so alternative tests such as single leg hop tests (SLHT), which better represent movement patterns, are preferred [22–24]. In addition, in the one-repetition maximum (1 RM) test, a field-based test that defines the maximum weight that can be lifted once, eccentric movements are often combined with concentric movements and better reflect natural movement in most sports and daily life activities [25, 26].

There are many studies in the literature that provide normative data specific to basketball [27], but it is seen that the data obtained mostly represent male athletes. In addition, vertical jump or 1RM squat tests were mostly used in studies examining the relationship between agility and strength in basketball players. However, as in the present study, no study was found in which isokinetic tests, SLHTs and 1 RM leg curl (LC), leg press (LP) and leg extension (LEX) tests were studied together with different agility tests. The aim of the present study was to investigate the relationship between agility and lower extremity strength in female basketball players. *The study hypothesized that there would be negative and significant correlations between agility performance and lower extremity strength parameters in basketball players.*

## Materials and methods

### Study design

The study had a cross-sectional study design with randomised measurements. Subjects visited the laboratory five times, including the *familiarisation session*. In the first visit, the participants were informed about the test protocols to be applied. At the same time, height, weight and body mass index (BMI) measurements were taken and SLHT, agility tests, 1 RM tests and isokinetic knee extension (Ex) and flexion (Flx) tests were demonstrated practically. Subjects were measured according to the tests they selected from the randomised practice cards. *The tests on the randomized practice cards were determined as follows: 1st Card: Single leg hop for distance (SL)*,* Triple leg hop for distance (THD)*,* Crossover hop for distance (CHD)*,* Medial side triple hop for distance (MSTH)*,* 90° Medial Rotation hop for distance (MRH). 2nd Card: 5-0-5 Agility Test (505)*,* Pro-agility Test (PA)*,* T Agility Test (T-test)*,* T Drill Hop Test (T). 3rd Card; 1 RM LP*,* 1 RM LEX*,* 1 RM LC. 4th Card; Concentric/concentric (Con/Con) isokinetic knee Ex and Flx tests at angular velocities of 60*,* 180*,* 240*,* and 300°/sec.*

Subjects were rested for 48 h after SLHTs, agility and isokinetic tests and 72 h after 1RM tests. SLHTs, 1 RM, isokinetic tests, 505, PA and T agility tests were performed on both right (R_S_) and left (L_S_) sides. Rest periods between tests were at least 5 min in SLHT and agility tests and at least 15 min in 1 RM tests. Isokinetic tests were performed according to the fixed protocol of the dynamometer, with only a minimum of 5 min rest between R_S_ and L_S_ transitions. After the minimum times for all tests were over, the subjects were asked if they were ready and the rest periods were extended up to 2 times. Before the tests, the subjects were given a general warm-up for the lower extremity muscles. During the measurements, participants were warned not to perform any exercise or physical activity and not to take stimulants such as caffeine until 48 h before the start of the tests. *All measurements were performed at the same time of day (14.00–16.00) and under similar environmental conditions (temperature ranging from 19 to 22 °C and humidity from 52 to 60%).*

The study was conducted in accordance with the ethical principles stated in the Declaration of Helsinki. Ethics committee approval was obtained from Ondokuz Mayıs University Clinical Research Ethics Committee (protocol no: 2022/315).

### Participants

Fifteen females aged 18–24 years (age 20.80 years, height 1.70 cm, weight 67.60 kg and BMI 23.20 kg/m²) participated in the study voluntarily (Table 1). The sample size of the study was determined by G*Power (version 3.1.9.6,* Germany) analysis (d = 0.8; α = 0.05; 1-β = 0.8) and the minimum number of subjects was 15.* The inclusion criteria for the study were to be actively playing basketball, to have an active training history of at least 5 years, and to have no previous lower extremity injury history. Volunteers who did not meet the inclusion criteria were excluded from the study. Voluntary Consent Form was obtained from the participants.

**Table 1:** Descriptive data of the subjects (n: 15)

	Mean	SD	Min.	Max.
Age (year)	20.80	1.82	18.00	24.00
Height (cm)	1.70	0.07	1.59	1.83
Weight (kg)	67.60	11.67	58.00	93.00
BMI (kg/m²)	23.21	3.23	19.36	30.04
Training Experience (years)	6.19	1.10	5.00	9.00

SD, standard deviation; Min., minimum; Max., maximum; BMI, body mass index

## Procedures

### Agility tests

#### T agility test (T-test)

After the participants were informed about the test, general warm-up procedures were applied. After the test start line was determined, photocells were installed in this area. A center cone was placed at a point 9.14 m (10 yards) from the starting line. Then one more cone was placed 4.57 (5 yards) metres to the right and left of the middle cone and the test area was prepared. During the test, the participant took position at the starting line and was warned that she could exit when ready. The photocells were switched on with the exit of the participant. The participant first ran quickly to the center cone and touched it. Immediately after touching the center cone, *the participant* slid sideways and touched the right cone. After touching the right cone, *she* quickly slid towards the leftmost cone and touched the left cone. Immediately after touching the cone on the left, the participant touched the cone in the center again by sliding sideways and then ran backwards and crossed the line where *she* started and stopped the photocell time. In side shifts and running backwards, the participant’s body was completely facing the opposite direction and did not turn in the direction of running [28].

### Pro-Agility test (5-10-5) (PA)

After participants were informed about the test, they performed general warm-up procedures. After warming up, the participant took a position on the starting line parallel to the direction in which she would run. The starting line was set as the center line and 2 more lines were found on either side of it at a distance of 4.57 (5 yards) metres. The time is started when the participant starts running from the starting line towards the line to her left. After running for 4.57 (5 yards) metres to the line on the left, the participant made a 180° turn by stepping on the turn line and ran quickly to the line on the far right for 9.14 (10 yards) metres. After reaching the line on the right, the participant stepped on the turn line in the same way. Then, she made a 180° turn, ran back the way and finished the test by sprinting to the center line and crossing it [29].

### 505 agility test (505)

0-m, 5-m and 15-m distances were determined in the test area. Cones were placed in the 0-m and 15-m zones and photocells were placed in the 5-m zone. Participants started to accelerate after exiting the 15-m zone. They started the photocells by passing through the 5-m zone and when they reached the 0-m zone, they turned back with a 180° turn and passed through the same photocell and stopped the time. The time before and after the 5-m turn was recorded with a photocell. The subjects performed a total of four performances, two with the right foot and two with the left foot [30].

### T-Drill hop test (T)

In preparation of the test area, photocells were placed across the start and a 10 ft (3.05 m) long straight line was drawn across this point. From the end of the 10 ft strip, a further 10 ft (1.52 m) straight strip was run 5 ft (1.52 m) to the right and 5 ft (5 m) to the left. In the execution phase of the test, the subject took their place at the starting point and started the photocells when they exited. The subjects started the test with the right foot and completed the course by bouncing 10 ft forward, 5 ft to the right, 10 ft to the left, 5 ft to the right and 10 ft backwards and stopped the photocells at the starting point. In the test started with the left foot, the participant first travelled to the left in the opposite direction and completed the test. Participants hopped with one foot only throughout the test. The participant’s body or feet faced the opposite direction throughout the test. Participants did not turn in these directions on the left and right tabs. Participants were given two rights for both the right and left foot in all tests and their best performance was recorded for each foot. In all tests, the test time was recorded in seconds [31].

### Isokinetic knee strength tests

In the study, knee Ex and Flx strengths of the participants were measured with Humac Norm (CSMI, USA) isokinetic dynamometer. Participants warmed up with the general warm-up protocol before starting the test. The seat, adapter, dynamometer and other settings of the dynamometer were made according to the fixed protocol suitable for the measurement of knee Ex and Flx strengths. Knee Ex and Flx strengths were measured at four different angular velocities: 60°/sec. and 180°/Sect. (4 repetitions, 15 s. rest, 5 repetitions), 240°/sec. and 300°/Sect. (4 repetitions, 15 s. rest, 15 repetitions) with a fixed protocol of con/con contractions with 30 s. rest between each velocity. Participants were informed about the number of repetitions remaining during the tests. And verbal support was given to the participants to increase motivation. Peak Torque values obtained in the measurements were recorded in Newton metres (Nm).

### Single leg hop tests (SLHT)

Participants were measured by SL, THD, CHD, MSTH and MRH tests from SLHTs. For these measurements, a tape measure of 6 m length and 15 cm width fixed to the ground was used. Participants started the test with their fingertips reset to the starting point of the jump line and the last point where their heels touched the ground was marked and the distance was recorded in cm. In SL, participants were instructed to stand on the leg to be tested and jump as far forward as possible and land with the same leg. In TH, participants were instructed to perform the same protocol as the SL test by performing three consecutive jumps. In CHD, participants were instructed to stand on the leg to be tested and jump to the farthest possible point 3 times in a row in different directions. In MSTH, participants were instructed to stand on the leg to be tested with the medial side of the foot vertical to the direction of the jump. After the participants jumped to the farthest point on the same leg three times in a row, the total distance was measured from the medial part of the foot in the starting position to the medial part of the foot at landing. In MRH, participants were instructed to stand on the leg to be tested with the medial side of the foot at the starting line. In the transverse plane, they were asked to jump on one foot, turn 90° in the air and turn their feet in the direction of the jump line during landing. Before the jump, participants were not allowed to turn their feet in the direction of the jump. The foot position vertical to the direction of the jump was carefully checked by the researchers. Landings in accordance with the procedure were considered successful for all jumps. Landing after jumping was confirmed when the participant had full control over the tested limb. Measurements were taken with the same methods for both legs. The test was repeated when the participant lost balance, touched the wall, or made additional bounces [23, 32, 33].

### One-Repetition maximum test (1 RM)

Participants were warned not to perform any strenuous activity or lower extremity resistance exercise for 48 h prior to the tests. Maximal dynamic strength measurements were performed using double-single leg LP, LEX, LC movements. Participants were informed about the tests and the movements to be performed. The exercises included in the study were performed on weight machines. Because the participants’ resistance exercise experience was quite limited and there was no homogeneity within the group. In this way, safety was ensured first and then the correct technique was applied. To ensure that the movements were performed with the correct technique, knee angles were determined with a ganyometer used manually by the researcher. Before the tests, the participants had a 10-minute dynamic warm-up.

After the general and special warm-up, the starting weight was determined by taking into account the answer to the question ‘What is your estimated 1 repetition maximal value?’. After the starting set, the researcher asked the participants if they could perform more lifts; if the answer was yes, the weight was increased and the participants performed one more set. First, subjects performed a set of 5–10 repetitions at 40–60% of their estimated maximal value. After a 3-minute active rest, subjects performed 5 repetitions at 60–80% of their estimated (perceived) maximal. After another 3 min of rest, subjects first had 3 RM and then 1 RM. A rest period of 2–3 min was given between the sets. The subjects were given a maximum of 5 trials for each movement. When the single repetition maximal values of the subjects could not be measured, maximal values were calculated from the 3 repetition set scores. A rest interval of at least 5 min was given between the movements and this period was extended for each subject until felt ready. The order of the movements was determined as LP, LEX, LC, taking into account the size and number of muscles active during the exercise, and each movement was first performed as double leg and then single leg. The researchers controlled the movement technique during the test and stopped the test if they thought the movement would harm the participants. The 3 repetition set was chosen because the sub-maximal load is safer for athletes who do not regularly lift weights and it is a reliable reference value for determining 1 RM [34].

### Statistical analysis

Statistical analysis of the study was performed in SPSS 22. package programme. Normality and equality of variance of the data were evaluated by Shapiro-Wilk, Q-Q plot and Levene tests, respectively. The data were found to be normally distributed. Independent and paired sample T-Test were used for pairwise comparisons and One-Way ANOVA test was used for three or more group comparisons. The relationships between variables were analysed using Pearson correlation analysis. In correlation analysis, > 0.30 was determined as weak correlation, 0.3–0.6 as moderate, 0.6–0.8 as high and 0.8-1.0 as excellent correlation [35]. The results were evaluated at *p* < 0.05 significance level.

## Results

**Fig. 1:**
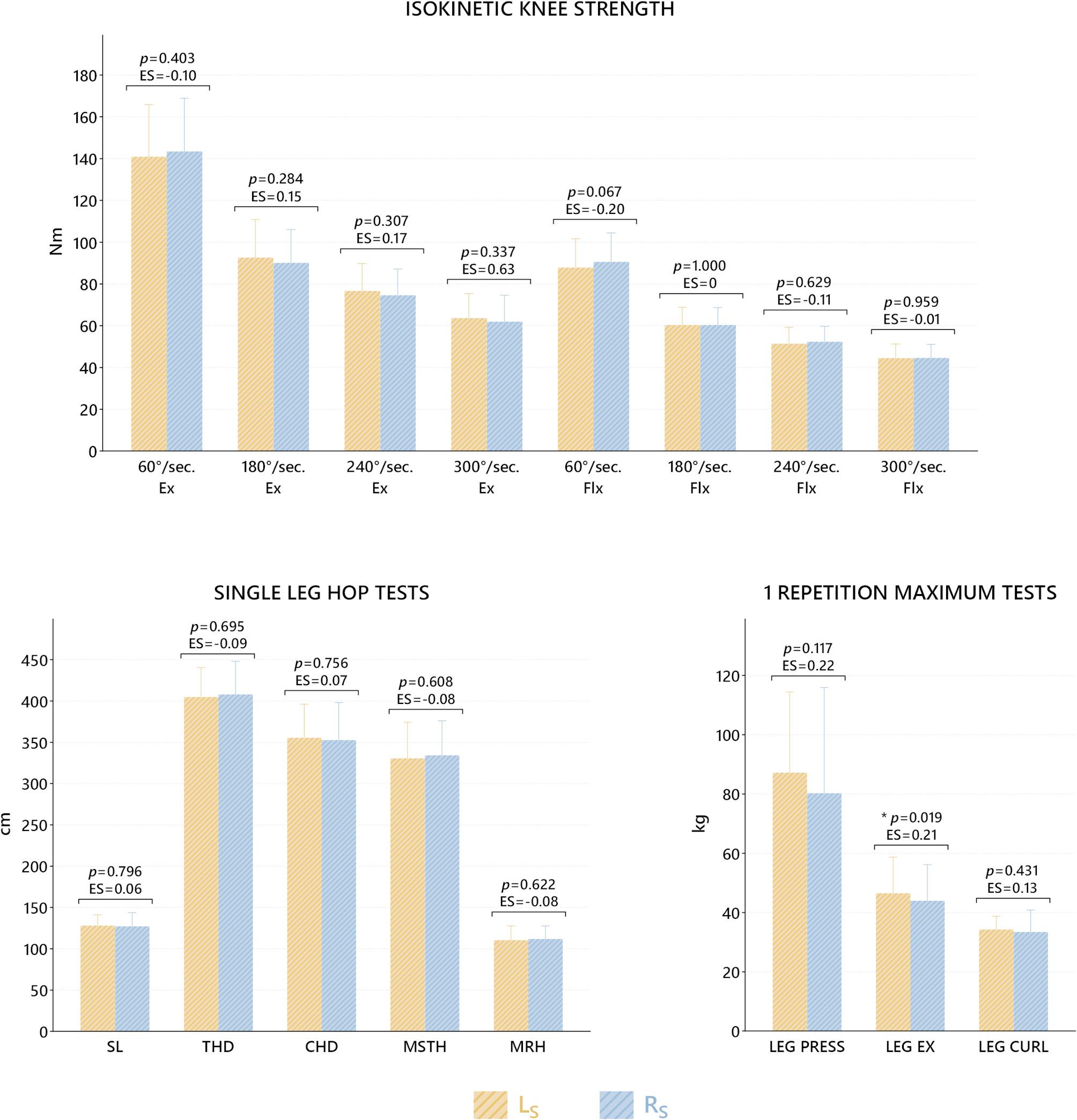
Comparisons of isokinetic tests, SLHTs and 1 RMs between R_S_ and L_S_

When the comparisons of R_S_ and L_S_ sides in isokinetic tests, SLHTs and 1 RM tests were evaluated statistically, a significant difference was found only in the LEX parameter from 1 RM tests (*p* = 0.019, 95% CI = 0.47–4.59) (Fig. 1).

**Fig. 2:**
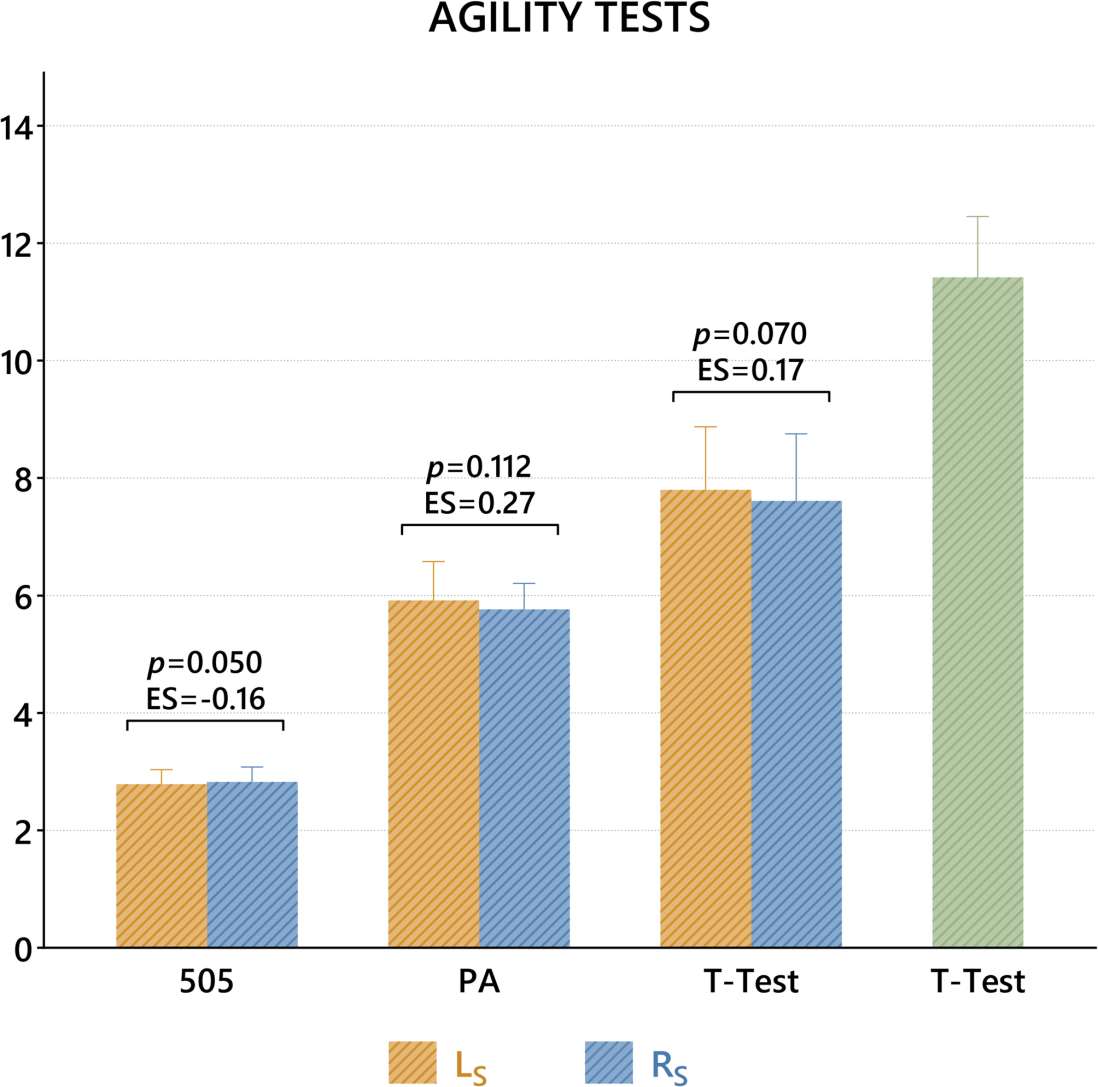
Comparison of agility tests between R_S_ and L_S_

When the results of agility tests on R_S_ and L_S_ sides were compared, there was no significant difference in the 505 (*p* = 0.050, 95% CI = 0.68–0.00), PA (*p* = 0.112, 95% CI= -0.04–0.34) and T (*p*=-0.070, 95% CI= -0.04–0.34) tests. Since T-Test results could not be evaluated in terms of R_S_ and L_S_, they are presented only as mean (Fig. 2).

**Fig. 3:**
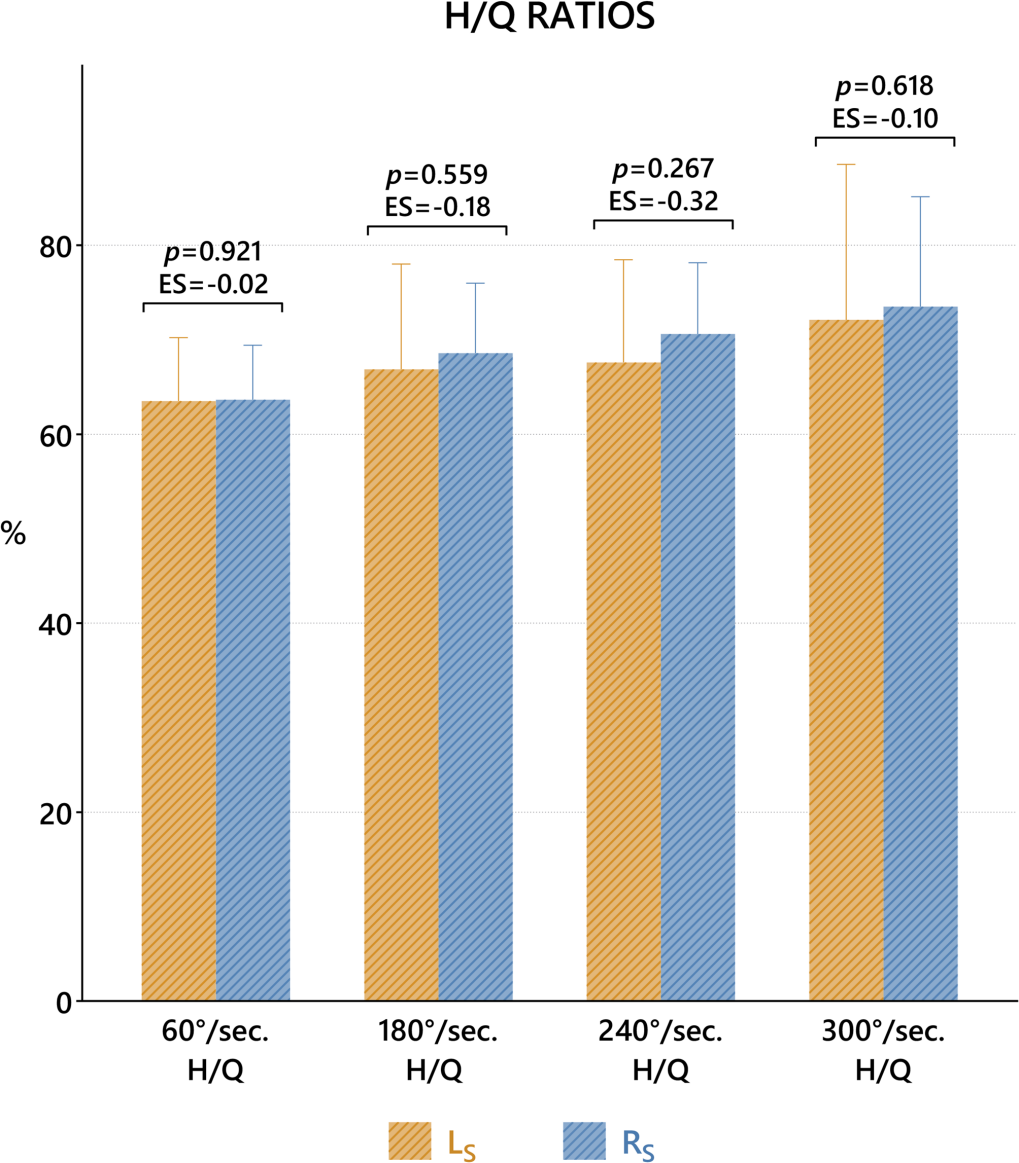
Comparison of H/Q ratios of isokinetic tests at different angular velocities

The H/Q ratios produced by isokinetic tests at different angular velocities on the R_S_ and L_S_ sides were compared. The findings showed that the R_S_ and L_S_ sides were statistically similar at angular velocities of 60, 180, 240 and 300°/sec (*p* > 0.05) (Fig. 3).

**Fig. 4:**
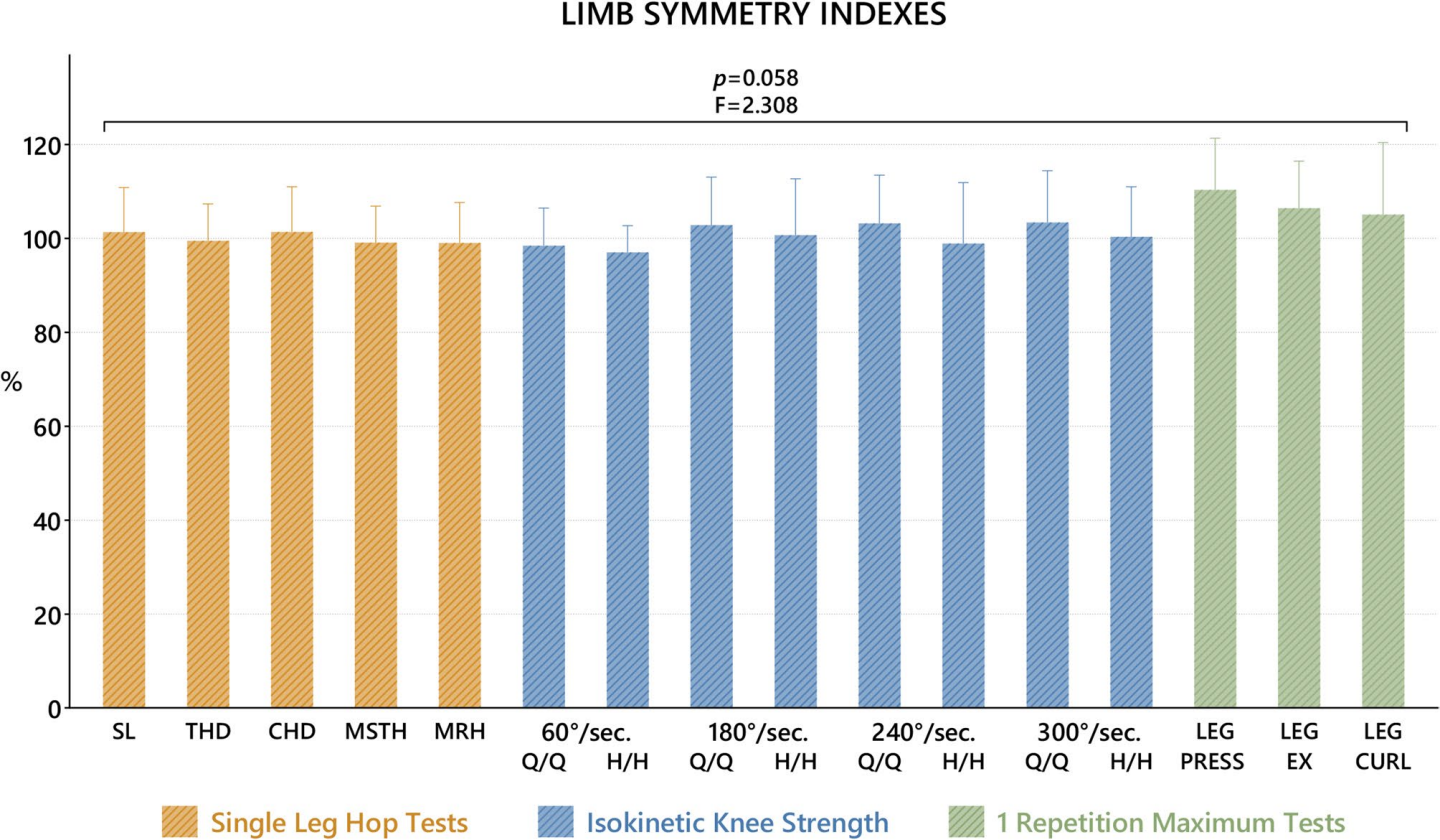
Comparison of LSIs in isokinetic tests, SLHTs and 1 RMs

LSIs calculated from the strength generated by the R_S_ and L_S_ sides in isokinetic tests, SLHTs and 1 RM tests were evaluated with the One-Way ANOVA test. When the results were evaluated, no statistically significant difference was found between all tests (*p* = 0.058) (Fig. 4).

**Fig. 5:**
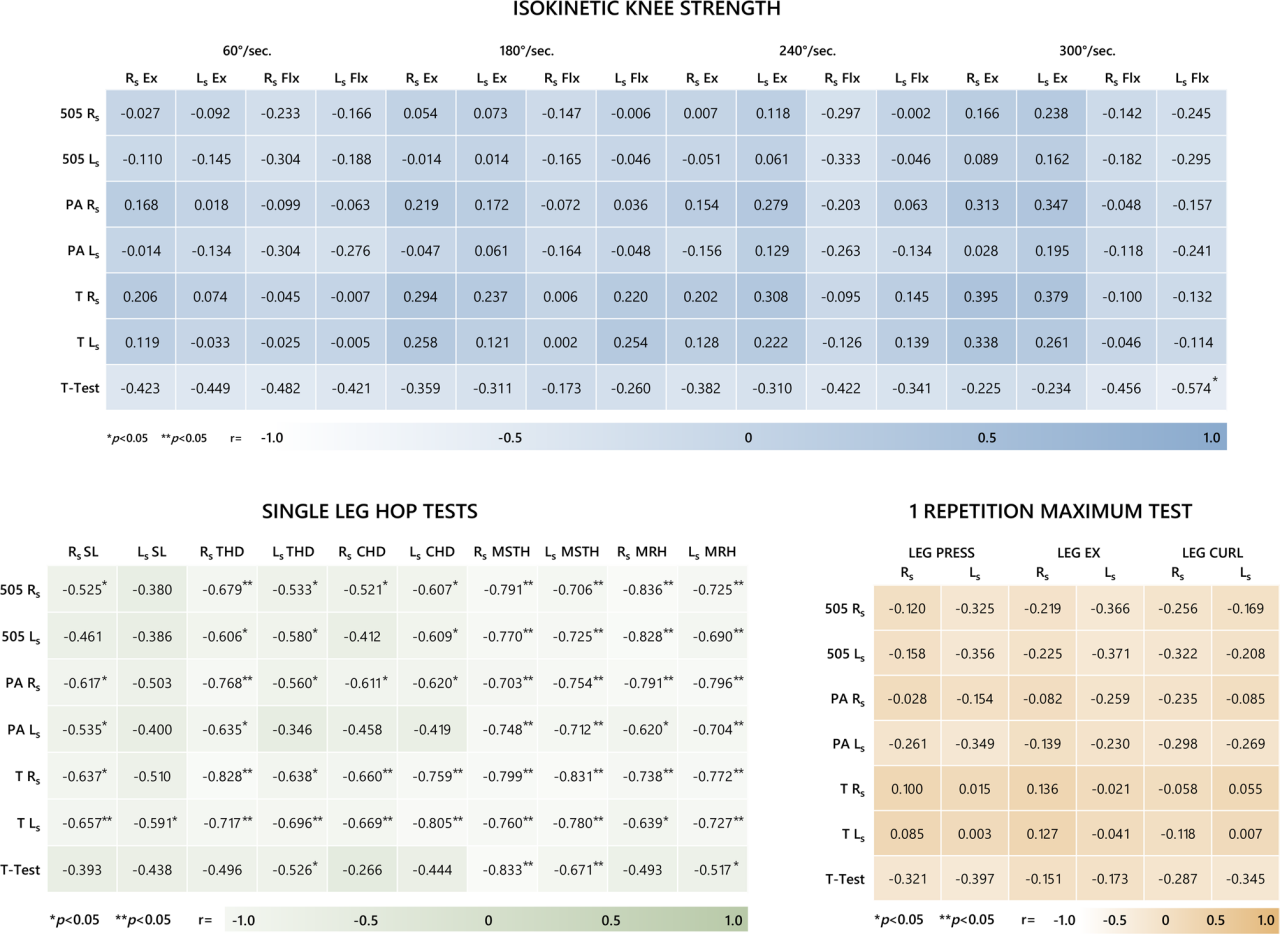
Correlations between agility tests and other test parameters

The relationships between the agility and strength test results were examined using the Pearson correlation test. The results showed that there was no significant correlation between agility tests and 1 RMs (*p* > 0.05). In addition, it was determined that there was a negative significant correlation between the T-test and L_S_ Flx at 300°/sec angular velocity (*r*=-0.574). When the correlations between SLHTs and agility tests were examined, it was seen that all agility tests and SLHTs had negative, moderate, high and excellent level significant correlations (*r*=-0.521 to *r*=-0.836) (Fig. 5).

## Discussion

In this cross-sectional study with randomised measurements, the relationship between agility and lower extremity strength in female basketball players was investigated. The main finding of the study was that agility tests had no statistically significant relationship with 1 RM tests, but there were negative, moderate and high level relationships with SLHTs. Similarly, isokinetic Flx strength results at an angular velocity of 300°/sec and T-Test showed a significant negative correlation. On the other hand, no significant difference was found in the isokinetic strength, SLHTs and agility tests on the R_S_ and L_S_ sides of the participants, while a significant difference was found in the LEX test among the 1 RM tests. Finally, it was observed that LSIs calculated from strength outputs had similar ratios in all tests.

In recent years, many studies have been conducted to investigate inter-limb strength asymmetries in order to protect athletes from injuries and evaluate performance parameters [36–38]. One study observed that inter-limb strength asymmetry negatively affected jumping, speed and change of direction performance [39]. Similarly, Maloney and Fletcher [40] found that strength asymmetry in healthy adults was associated with poor performance on agility tests. Therefore, the assessment of lower limb strength potential and possible strength asymmetry on both R_S_ and L_S_ sides is critical for maximum agility performance in basketball [13]. In the present study, isokinetic, SLHT and 1 RM test results on the R_S_ and L_S_ sides showed that there was no significant difference between both extremities. This result is similar to previous studies examining the effect of strength outcomes on agility [27, 36]. One study suggested that the lack of strength differences between the extremities will have a positive effect on agility performance, since sudden changes of location-direction in basketball with the ball (such as crossover) or without the ball (such as V cut or L cut) are performed on both sides [21]. Similarly, the 505, PA and T agility tests used in our study were applied to the R_S_ and L_S_ sides separately and the results were similar on both sides. This suggested that subjects benefited from similar SSC on both the R_S_ and L_S_ sides.

Lower limb strength refers to the strength produced by the leg muscles (especially the quadriceps, hamstring and gluteal muscles) during multidirectional joint movements and is one of the key components of agile movements supported by the anaerobic energy system [41]. The hypothesis of the present study was based on this scientific knowledge and the correlations between lower extremity strength parameters and agility confirmed the hypothesis. Because the correlation analyses showed that the results obtained in all SLHTs were negatively correlated with agility tests at moderate, high or excellent levels. In other words, the increase in jump distances in SLHTs will lead to a decrease in agility test times and therefore a more efficient agility performance. This result in our study showed that SLHTs have the ability to represent short-term and high-intensity movements such as sudden change of direction used during competition, and there were other studies in the literature confirming this result [42, 43]. A study showed that agility performance improved with increasing lower extremity strength in futsal athletes [44]. In a similar study, Papla et al. [45] found that lower extremity strength and power parameters were significantly related to agility performance in basketball players. On the other hand, the relationship analyses showed that 1 RM and isokinetic test results were not significantly correlated with agility tests. When this result was evaluated in terms of strength types, 1 RM and isokinetic tests are more representative of maximal strength production [46]. Therefore, it is considered that these tests are not functional and cannot represent real-time agility skills. However, the fact that only the isokinetic Flx strength at an angular velocity of 300°/sec has a significant negative correlation with the T-Test explains that joint movements in sudden changes in direction are similar to agility actions in terms of application since they are performed at high speed in a very short time. In addition, lower extremity Flx strength is an important biomechanical factor that directly affects acceleration and deceleration cycles in athletes. Because this strength refers specifically to the strength of the muscle groups that perform Flx movements of the knee and hip joints (for example, hamstrings and iliopsoas) [47]. However, it is not only strength that is important, but also the ability to apply this strength quickly. Therefore, power, which is the combination of strength and speed, is an important factor in determining sprint and change of direction performance [48]. A study reported that there was a significant correlation between lower limb Flx strength at 300°/sec angular velocity and agility performance on footballers, but this result was not reached at 60°/sec angular velocity [49].

On the other hand, unilateral isokinetic, SLHT and 1 RM test outputs provide information about bilateral and ipsilateral asymmetric strength ratios in the lower extremities [50–52]. These asymmetric strength ratios are an important criteria for injury incidence, especially in the athletic population [53]. When isokinetic lower extremity strength outputs were examined in the current study, it was found that ipsilateral hamstring/quadriceps (H/Q) strength ratios were within the normal range at all angular velocities and increased as angular velocities increased [54, 55]. In addition, bilateral and ipsilateral LSI rates calculated in all strength tests were in the range of 10–15% [25], indicating that there was no negative effect on the performance of the subjects. This result is similar to many studies conducted in the athletic population [56–58]. In addition, maintaining ipsilateral and bilateral strength ratios in the lower limb within optimal limits not only reduces the incidence of injury, but also contributes positively to agility performance by increasing the efficiency of neural impulses to agonist and antagonist muscles and accelerating the transition between deceleration and acceleration actions [5, 16].

Although the present study had advantages when compared with the literature, it had several limitations such as working with the minimum subject group obtained in the power analysis, not comparing gender and age factors, not examining the gluteal muscles and plantar Flx strength outputs, which are thought to have a significant effect on lower extremity strength potential. In addition, deformities in the metaphyseal and epiphyseal regions of the tibia, which may have a negative effect on the lower extremity strength potential of the participants, were excluded. Therefore, there is a need for supportive studies examining the relationships between strength and agility by considering deformities that may be directly related to the lower extremity. In addition, increasing and diversifying the number of participants will contribute to a more detailed examination of the relationship between lower extremity strength and agility. *Finally*,* similar studies based on the positions of basketball players may give more specific results.*

## Conclusions

Our study showed that lower extremity functional strength parameters had a significant relationship with agility skill in female basketball players. In addition, this relationship was seen in all SLHTs but not in 1 RM tests and isokinetic test outcomes except for Flx strength at 300°/sec angular velocity. Finally, LSI ratios, which give important results for injury tendencies, were found to be within normal ranges in all strength tests.

## Data Availability

Data supporting the findings of this study are available through the corresponding author, but restrictions apply to the availability of these data used for the current study and are therefore not publicly available. However, data are available from the corresponding author (akerim.yilmaz@omu.edu.tr) upon reasonable request.

## Abbreviations

1RM: One-Repetition Maximum Test
505: 505 Agility Test
BMI: Body Mass Index
CHD: Crossover Hop for Distance
Con/Con: Concentric/Concentric
Ex: Extension
Flx: Flexion
H/Q: Hamstring/Quadriceps
LC: Leg Curl
LEX: Leg Extension
LP: Leg Press
L_S_: Left Side
LSI: Limb Symmetry Indexes
Max.: Maximum
Min.: Minimum
MRH: 90° Medial Rotation Hop for Distance
MSTH: Medial Side Triple Hop for Distance
Nm: Newton metres
PA: Pro-Agility Test
R_S_: Right Side
SD: Standard Deviation
SL: Single Leg Hop for Distance
SLHT: Single Leg Hop Tests
SSC: Stretch-Shortening Cycle
T: T Drill Hop Test
TH: Triple Leg Hop for Distance
T-test: T Agility Test
